# Mouse ICM Organoids Reveal Three-Dimensional Cell Fate Clustering

**DOI:** 10.1016/j.bpj.2018.11.011

**Published:** 2018-11-16

**Authors:** Biena Mathew, Silvia Muñoz-Descalzo, Elena Corujo-Simon, Christian Schröter, Ernst H.K. Stelzer, Sabine C. Fischer

**Affiliations:** 1 Physikalische Biologie, Fachbereich Biowissenschaften, Buchmann Institute for Molecular Life Sciences, Goethe-Universität Frankfurt am Main, Frankfurt am Main, Germany; 2 Department of Biology and Biochemistry, University of Bath, Bath, United Kingdom; 3 Instituto Universitario de Investigaciones Biomédicas y Sanitarias, Universidad Las Palmas de Gran Canaria, Las Palmas de Gran Canaria, Spain; 4 Department of Systemic Cell Biology, Max-Planck-Institute of Molecular Physiology, Dortmund, Germany

## Abstract

During mammalian preimplantation, cells of the inner cell mass (ICM) adopt either an embryonic or an extraembryonic fate. This process is tightly regulated in space and time and has been studied previously in mouse embryos and embryonic stem cell models. Current research suggests that cell fates are arranged in a salt-and-pepper pattern of random cell positioning or a spatially alternating pattern. However, the details of the three-dimensional patterns of cell fate specification have not been investigated in the embryo nor in in vitro systems. We developed ICM organoids as a, to our knowledge, novel three-dimensional in vitro stem cell system to model mechanisms of fate decisions that occur in the ICM. ICM organoids show similarities to the in vivo system that arise regardless of the differences in geometry and total cell number. Inspecting ICM organoids and mouse embryos, we describe a so far unknown local clustering of cells with identical fates in both systems. These findings are based on the three-dimensional quantitative analysis of spatiotemporal patterns of NANOG and GATA6 expression in combination with computational rule-based modeling. The pattern identified by our analysis is distinct from the current view of a salt-and-pepper pattern. Our investigation of the spatial distributions both in vivo and in vitro dissects the contributions of the different parts of the embryo to cell fate specifications. In perspective, our combination of quantitative in vivo and in vitro analyses can be extended to other mammalian organisms and thus creates a powerful approach to study embryogenesis.

## Introduction

Understanding preimplantation development is key to an improved success of pregnancies in mammals. In humans, up to 40% of embryos die during the development from a fertilized egg to a blastocyst ready for implantation into the uterus (1). In mice, i.e., in the common model system for mammalian development, the blastocyst stage lasts from embryonic day 3.0 (E3.0) to E4.5 after fertilization. During this phase, the inner cell mass (ICM) segregates into the epiblast (embryo precursors; Epi) and the primitive endoderm (yolk sac precursors; PrE). The ICM is enclosed by the trophectoderm (TE). The transcription factors NANOG and GATA6 are the earliest markers for Epi or PrE fate, respectively, and have been identified as key transcription factors to support these emerging cell fates (2). Before cells in the ICM adopt different fates, ICM cells coexpress NANOG and GATA6 (3).

At the mid-blastocyst stage, ICM cells express either NANOG or GATA6, resulting in a mutually exclusive expression often referred to as a salt-and-pepper pattern (3, 4, 5). Previous studies have shown that FGF/ERK-signaling is required for the emergence of Epi and PrE cells, marked by mutually exclusive expressions of NANOG and GATA6 (2). FGF/ERK-signaling is activated by FGF4 (fibroblast growth factor-4) secreted from NANOG expressing cells. Existing models, which imply local FGF4-signaling in combination with cell division and cellular organization, result in a specific three-dimensional arrangement of cells with discrete fates (6, 7, 8). The pattern is not necessarily equivalent to a random intermingling of GATA6-positive and NANOG-positive cells, i.e., the cells do not exhibit the spatial randomness referred to as a salt-and-pepper pattern. The exact spatial arrangement with which cell fates emerge in the ICM has not been determined experimentally. However, to obtain the full benefit from mathematical models with respect to mechanistic insight, quantitative characterization of the patterns is indispensable. Because complex three-dimensional patterns cannot be analyzed visually, the quantitative analysis must distinguish between a random intermingling of cells and an alternatively organized pattern. Although FGF4 is known to be important for PrE specification, instructive signals for Epi specification have not been identified.

In the late blastocyst, GATA6-positive cells are sorted to the ICM surface facing the cavity and form the PrE. During the initiation of implantation, NANOG is downregulated (9, 10). Whether NANOG inhibition depends on FGF4 expression or is triggered through PrE differentiation is yet unknown (10). Subsequently, laminins are secreted by TE and PrE cells to assemble a basement membrane at the interface to the Epi (11). The PrE further develops into two cell lineages: the parietal endoderm (PE) and the visceral endoderm (VE) (12). The VE cells remain attached to the Epi, whereas the PE cells migrate along the trophectodermal basement membrane (13).

Studies of mammalian embryogenesis rely heavily on mouse embryos. Pregnant females are culled, and between 6 and 15 embryos are collected. During the last decades, mouse embryonic stem cells (mESCs) have been used to study lineage specification and commitment during embryonic development. They are extracted from a single mouse, which addresses ethical issues. mESCs are capable of spontaneously organizing into three-dimensional aggregates (embryoid bodies, EBs), which have been employed as in vitro models for differentiation, including the formation of an outer endodermal layer, an inner Epi core and a basement membrane (13, 14). The combination of EBs with computational rule-based modeling has previously been implemented to predict emergent spatial patterns during cell fate transitions (15). However, the spatial arrangement of PrE-like cell types over the course of development has not been quantitatively analyzed in any EB system.

In this study, we took advantage of an mESCs-based system, which allows differentiation of PrE-like cells on a timescale that resembles the emergence of the PrE in the embryo (16). The proteome of PrE-like cells that differentiate in this ESC system resembles that of embryo-derived XEN cells, the tissue culture equivalent of the embryonic PrE (17). We demonstrate that three-dimensional aggregates of these cells recapitulate core aspects of cell fate decisions made in mouse ICMs during preimplantation. This includes the mutually exclusive expression of NANOG and GATA6, sorting and evidence for PE and VE development. These new three-dimensional stem cell aggregates approximate the timescale of events and the cell identities of mouse ICMs more closely than EBs. Hence, we term them “ICM organoids.”

To characterize the spatiotemporal patterns of cell fate decisions in the embryos and the ICM organoids, we combined our previously developed quantitative image-based neighborhood analyses (18, 19) with rule-based modeling. We focus on the levels of fate markers expressed by a cell and its neighbors and the number of neighboring cells as the features of interest in the cell neighborhood. We obtain quantitative agreement between our ICM organoids and mid- or late blastocysts’ ICMs, respectively. The analysis of the cellular neighborhood through computational rule-based modeling revealed a so far unknown local clustering in the spatial distribution of NANOG- and GATA6-positive cells. The comparison of the in vitro and in vivo data suggests that cell fate decision and patterning in the ICM during preimplantation are not primarily dependent on the TE. It seems more appropriate to assume that the TE provides a mechanical constraint.

Our qualitative and quantitative characterization of the newly proposed ICM organoids provides a, to our knowledge, new perspective on the mechanisms of Epi versus PrE differentiation in mammals.

## Materials and Methods

### ESC culture

mESCs used in this study (tet::GATA4-mCherry and tet::GATA6) have an inducible GATA4 or GATA6 transgene based on the KH2 mESC line (16).

Cells were cultured on 0.1%-gelatin-coated cell culture flasks in Glasgow Minimum Essential Medium (Gibco, Waltham, MA) supplemented with 10% fetal bovine serum (Labtech, Marietta, GA), 2 mM glutamax (Gibco), 1 mM sodium pyruvate (Sigma-Aldrich, St. Louis, MO), 0.1 mM nonessential amino acids (Sigma-Aldrich), 0.1 mM 2-mercatoethanol (Gibco), and 5 × 10^5^ U/mL leukemia inhibitory factor (Merck Millipore, Burlington, MA).

Medium was changed daily, and cells were passaged when a confluency of 70–80% was reached. Cells were detached with 1 mL 0.05% trypsin-EDTA (Gibco) and resuspended in 5 mL culture medium. Cell suspension was gently homogenized by pipetting and centrifuged at 1000 rotations per minute for 3 min. Subsequently, cells were washed with 5 mL culture medium. Cells were maintained in their respective medium at 37°C and 5% CO_2_.

### ESC differentiation and ICM organoid formation

For two-dimensional cell culture, Thermanox cell-culture-treated plastic coverslips (Ø 25 mm; ThermoFischer, Waltham, MA) were situated in the wells of six-well culture dishes. 0.1% gelatin in phosphate-buffered saline (PBS) was added in each well to create a coated surface. The gelatin was allowed to dry overnight. Cells were seeded on the gelatinized coverslips at a density of 75 × 10^3^ cells and cultured for 3 days in cell culture medium containing 1 *μ*M PD0325901 (R&D Systems, Minneapolis, MN). Medium was exchanged daily. After 3 days, the induction of GATA4 or GATA6 expression was achieved by the addition of 500 ng/mL doxycycline (Sigma-Aldrich). Doxycycline (dox) was removed after 6 h. Cells were further kept in cell culture medium, which was changed daily.

For aggregates, two T-25 flasks were seeded with 45 × 10^4^ cells in cell culture medium containing 1 *μ*M PD03. Medium and PD03 were replaced every day. At the fourth day, culture medium of one flask was replaced by medium containing 500 ng/mL dox. After 6 h, cells were detached, and ICM organoids were formed as described in the following:

Aggregates from 200 cells with tet::GATA4: 96-well culture dishes were coated with 50 *μ*L 1% low-melt agarose in PBS to form concave wells. All wells were preincubated with 50 *μ*L medium containing serum and leukemia inhibitory factor (LIF) for 6 h at 37°C and 5% CO_2_. Per well, 200 cells were seeded in 100 *μ*L culture medium. Well plates were centrifuged at 1000 rotations per minute for 3 min to concentrate the cells in the middle of the well. Plates were kept undisturbed at 37°C and 5% CO_2_.

Aggregates from 50 or 200 cells with tet::GATA6: aggregates were formed after a 6 h dox induction by seeding 50 or 200 cells in each well of a U-bottom 96-well plate. They were not centrifuged and left undisturbed for 24 or 48 h.

### Immunostaining

Two-dimensional cell culture samples were fixed with 4% paraformaldehyde and subsequently blocked with blocking solution (1% bovine serum albumin, 0.1% Triton) for 60 min at room temperature. Samples were stained with anti-NANOG and anti-GATA6 antibody (1:200; R&D systems) overnight at 4°C. The secondary antibodies were an anti-rat Alexa Fluor 488 antibody (1:400; Invitrogen, Carlsbad, CA), anti-goat Alexa Fluor 647 antibody, and 1 *μ*g/mL DAPI (Thermo Scientific) for 1 h at room temperature.

Immunofluorescence staining of three-dimensional aggregates (tet::GATA4) in toto was performed according to Smyrek and Stelzer (20). In brief, aggregates were fixed with 4% paraformaldehyde and then permeabilized with 0.3% Triton X-100 in PBS for 30 min at room temperature. Subsequently, free binding sites were blocked with 0.1% bovine serum albumin, 0.2% Triton, 0.05% Tween-20, and 10% fetal bovine serum for 1 h at room temperature. The primary antibodies were anti-NANOG and anti-GATA6 (1:200; R&D Systems), anti-ZO-1 (1:200; Thermo Fischer), anti-caspase3 p17 (1:200; Cell Signaling, Danvers, MA), and anti-laminin (1:200; Sigma-Aldrich) and were incubated overnight at 37°C. The secondary antibodies were anti-rat Alexa Fluor 488 (1:400; Invitrogen), anti-goat Alexa Fluor 647 (1:400; Invitrogen), anti-rabbit Alexa Fluor 568 (1:400; Molecular Probes, Eugene, OR), anti-rabbit Alexa Fluor 559 (1:400; Invitrogen), and anti-rabbit Alexa Fluor 488 (1:400; Molecular Probes) and were incubated for 4 h at 37°C. Cell nuclei were counterstained with 1 *μ*g/mL DAPI (Thermo Scientific). Aggregates of tet::GATA6 cells were incubated at 4°C overnight. The primary antibodies were anti-GATA4 (1:200; Santa Cruz, Dallas, TX) and anti-SOX17 (1:200; R&D systems). The secondary antibodies were anti-rabbit Alexa Fluor 568 (1:500; Invitrogen), anti-goat Alexa Fluor 647 (1:500; Invitrogen), and anti-goat Alexa Fluor 568 (1:500; Invitrogen).

### Image acquisition

Fixed samples were mounted on 24 × 60 mm glass coverslips (Menzel Gläser #1) with Mowiol for tet::GATA4 cells or with Vectashield for tet::GATA6 cells. They were imaged with a Zeiss laser scanning confocal fluorescence microscope LSM780 or LSM880 (Zeiss, Oberkochen, Germany). Samples were imaged along the entire *z* axis with 0.33 *μ*m spacing for the two-dimensional culture or 1.28 *μ*m spacing for tet::GATA4 aggregates using a Plan-Apochromat 63×/1.40 oil immersion objective. Tet::GATA6 aggregates were imaged with a 1 *μ*m spacing using a Plan-Apochromat 40×/1.3 oil immersion objective. Images were acquired using 633, 561, 488, and 405 nm lasers.

### Image and data analysis

We performed three preprocessing steps on the image stacks of the nuclei channel. First, we applied the Fiji function “Enhance contrast” (settings: saturated 0.4%, normalize, equalize histogram, process all, and use stack histogram). Then, we used the Fiji function “subtract background” with a radius of 50 pixel. Finally, we used the dark elliptic features (21) as markers for filling extended minima in the image and applied a Gaussian filter of range 2.

For segmentation and feature extraction, we applied our previously developed multiscale image analysis pipeline (18). Parameter values were used as summarized in Table S1. The code and the user interface are available online (https://www.physikalischebiologie.de/downloads). In brief, the pipeline combines three-dimensional nuclei segmentation with topological and graph theoretical approaches to obtain quantitative measures of the cell nuclei, the local cell neighborhood, and the whole ICM organoid. The result is a cell graph in which vertices represent cells and edges represent the cell’s neighborhood. We used the Delaunay cell graph to approximate which cells are in physical contact. Two vertices are connected by an edge if the Euclidean distance between them is less than 30 *μ*m and the edges are part of the Delaunay triangulation of the vertices.

We obtained the fluorescence intensity values for NANOG and GATA6 for each cell and its neighbors. Applying a previously established *k*-means clustering approach (22) to the NANOG and GATA6 levels, we separated the cells into the four populations: double negative (DN; low NANOG, low GATA6), NANOG+/GATA6− (N+/G−), NANOG−/GATA6+ (N−/G+), and double positive (DP; high NANOG, high GATA6).

ICM organoid data was compared to the data by Saiz et al. (22) for mouse embryos, which is freely available (downloaded from Github on 30/11/2016). The data sets are provided as lists that contain, inter alia, the position of the nuclei and the cell population classification (TE, DN, N+/G−, N−/G+, DP). Based on these data, we calculated the Delaunay cell graphs for the cells of the inner cell mass. We applied a threshold of 30 *μ*m for the edges.

For the salt-and-pepper simulations, we generated four artificial patterns of expression level distribution. We used the cell nuclei positions of three 1-day-old ICM organoids (1755 cells in total) and simulated four artificial patterns for each.

Random pattern: we extracted the distribution of NANOG or GATA6 levels, respectively, and we randomly assigned expression levels to the cells of the simulated ICM organoids. Cells were separated into positive and negative cells based on the thresholds for NANOG or GATA6, obtained during the preprocessing of the data. Finally, we used the cell graph to determine the neighborhood composition.

Period two pattern: for each simulated ICM organoid, we generated a pattern in which positive cells have only negative neighbors (23).

Nearest neighbor pattern: one cell is assigned a negative state. The nearest neighbor is determined based on the Euclidean distance between the two nuclei centroids. This nearest neighbor is assigned a positive state. This was iterated until all cells were assigned a state.

Local clustering pattern: one cell is randomly assigned a positive or negative state. The nearest neighbor is determined based on the Euclidean distance between the two nuclei centroids. This nearest neighbor is assigned the same state as the previous cell with a given probability *p*. This was iterated until all cells were assigned a state. We varied *p* between 0.1 and 0.9 in steps of 0.1. Based on the mean deviation of the simulated from the experimental data, we found that the results for *p* = 0.9 matched the experimental data best.

We extended our simulations to generate patterns of four cell populations. We established the NANOG and GATA6 identities independently for each cell according to one of the patterns above and combined the information to obtain the population type of the cell, i.e., DN, DP, N+/G−, or N−/G+. Hence, if, e.g., we combined local clustering for NANOG with a period two pattern for GATA6, we used the approach for the local clustering pattern to set the NANOG expression (positive or negative) and the period two patterning rule for the GATA6 expression (positive or negative) for each cell in the simulated ICM organoids. The population type of a cell is then the combination of its NANOG and GATA6 expression setting.

For statistical comparison, we used the Wilcoxon-Mann-Whitney test and confidence intervals, both with Bonferroni correction for multiple testing if applicable. To determine the simulated pattern that provides the closest fit to the experimental data, we calculated the effect size as the relative deviation of the simulated data from the experimental data, i.e., $\left(\bar{s}-\bar{d}\right)/\bar{d}$, where $\bar{s}$ is the mean of the simulated data and $\bar{d}$ the mean of the experimental data.

Unless otherwise stated, the image and data analysis methods were implemented in Mathematica 11.1 (Wolfram Research, Champaign, IL).

## Results

### ICM organoids show key phases of preimplantation development: Mutually exclusive expression and sorting

**Figure 1:**
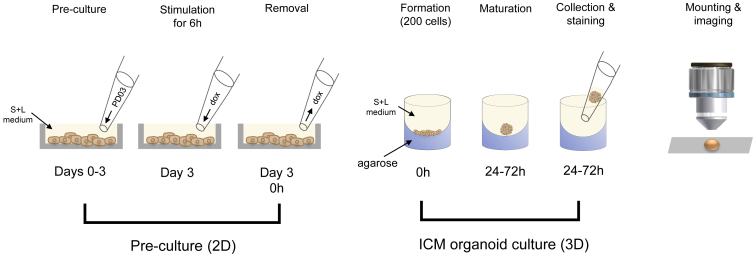
Generation and imaging pipeline for aggregates of mESCs (ICM organoids). Mouse tet::GATA4 ESCs were pre-cultured for 3 days in medium containing serum and LIF (S + L) and PD0325901 (PD03). At day 3, cells were stimulated with dox for 6 h to induce PrE differentiation. After dox removal, 200 cells were seeded in microwell plates coated with 1% low melt agarose. Aggregates were formed in medium containing S + L and were kept undisturbed. After 24, 48, and 72 h, aggregates were collected, stained, mounted, and imaged. To see this figure in color, go online.

Dynamic gene expression patterns are hallmarks of lineage specification in the ICM of the mouse blastocyst. To investigate the spatial organization of these events in a simplified yet three-dimensional setting, we established a simple experimental system that recapitulates the phases of the cell fate decisions that take place in the ICM. We formed three-dimensional aggregates from mESCs that carry a dox-inducible GATA4-FLAG transgene (16). Upon collection, the aggregates were fixed, immunolabeled, and imaged with a confocal microscope along their *z* axis (Fig. 1). Endogenous GATA6 expression was used to monitor differentiation events in individual cells over time. Transient expression of dox-inducible GATA4-FLAG instead of GATA6-FLAG was used because according to previous studies, it has the same activity in inducing PrE-like differentiation but can be expressed at higher concentrations from dox-inducible promoters and thereby increases the proportion of differentiating cells (16, 24, 25). As controls, we formed aggregates of uninduced cells.

**Figure 2:**
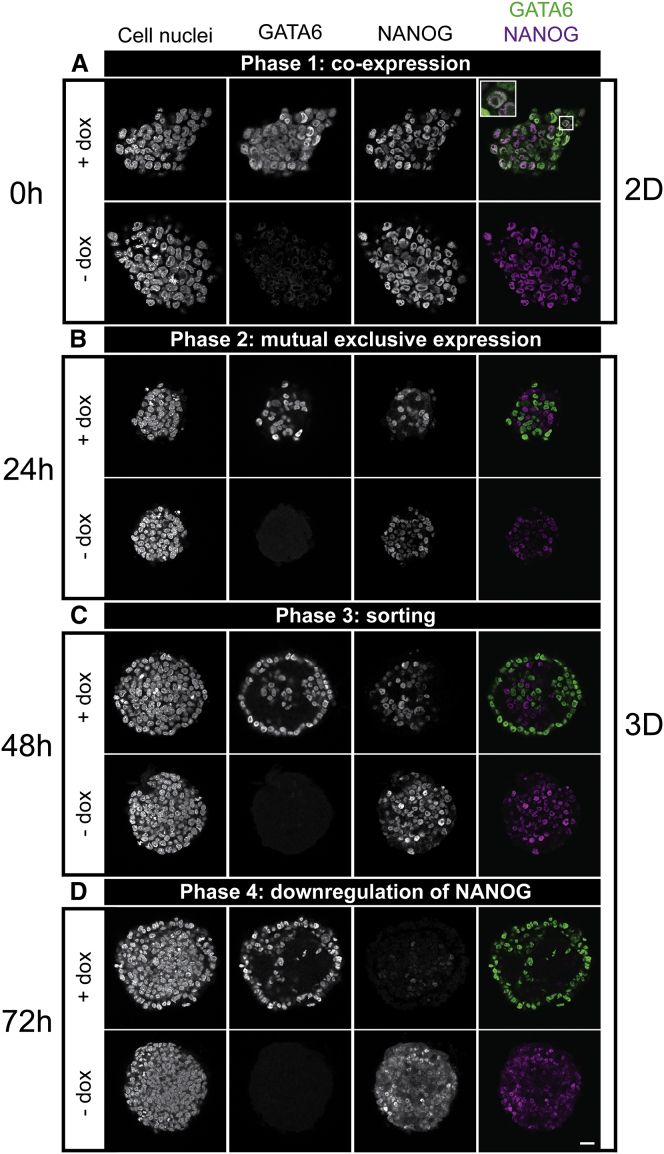
ICM organoids mimic the mouse ICM. Mouse tet::GATA4 ESCs were stimulated for 6 h with doxycycline (+ dox) to induce PrE differentiation. Cells that were not stimulated with dox (− dox) served as control. (*A*) After removal of dox, GATA6 and NANOG were coexpressed in cells of the two-dimensional monolayer of mESCs (see *box* for a magnified section of a coexpressing cell). (*B*) 24 h after ICM organoid formation, GATA6 and NANOG were mutually exclusively expressed within the ICM organoids. (*C*) After 48 h, GATA6-positive cells arranged at the rim of the ICM organoids. (*D*) After 72 h, NANOG was downregulated. Images show a single slice from the aggregate’s center. Microscope: Zeiss LSM780; objective: 63×/1.40 oil; scale bars, 20 *μ*m. To see this figure in color, go online.

Upon dox removal, endogenous GATA6 and NANOG were coexpressed in each cell of the monolayer (Fig. 2 *A*, *top row*). One day after aggregation, a mutually exclusive expression of GATA6 and NANOG arose in the cells (Fig. 2 *B*, *top row*). After 48 h, GATA6-positive cells formed a single cell layer that enclosed the aggregates, whereas NANOG-positive cells clustered in the inner core of the aggregates (Fig. 2 *C*, *top row*). The outer cell layer, reminiscent of an epithelium, displayed an ordered arrangement of the cell nuclei around the inner cells. Few GATA6-positive cells were also found in the core of the aggregates. The orthogonal views along *xz* and *yz* show that aggregates have a spherical shape after 48 h (Fig. S1). Mutually exclusive expression of NANOG and GATA6 as well as sorting also occurred in two-dimensional cultures (Fig. S2; (16)). After 3 days, NANOG expression was downregulated within the aggregates, reminiscent of the situation in the ICM in late mouse blastocysts just before implantation (Fig. 2 *D*; (9)). The decrease in NANOG levels was not due to apoptosis. Only marginal cleaved caspase activity was detected in the center of aggregates, not associated with dox-treatment and PrE-like differentiation (Fig. S3). Interestingly, NANOG is downregulated after 3 days, even though aggregates were still cultured in medium containing LIF. This suggests that an interaction between PrE-like and Epi-like cells might be involved in Epi maturation. None of the processes were observed in the control aggregates (Fig. 2, *bottom rows*). Consequently, PrE-like fate and Epi-like maturation do not arise spontaneously in our culture conditions.

**Figure 3:**
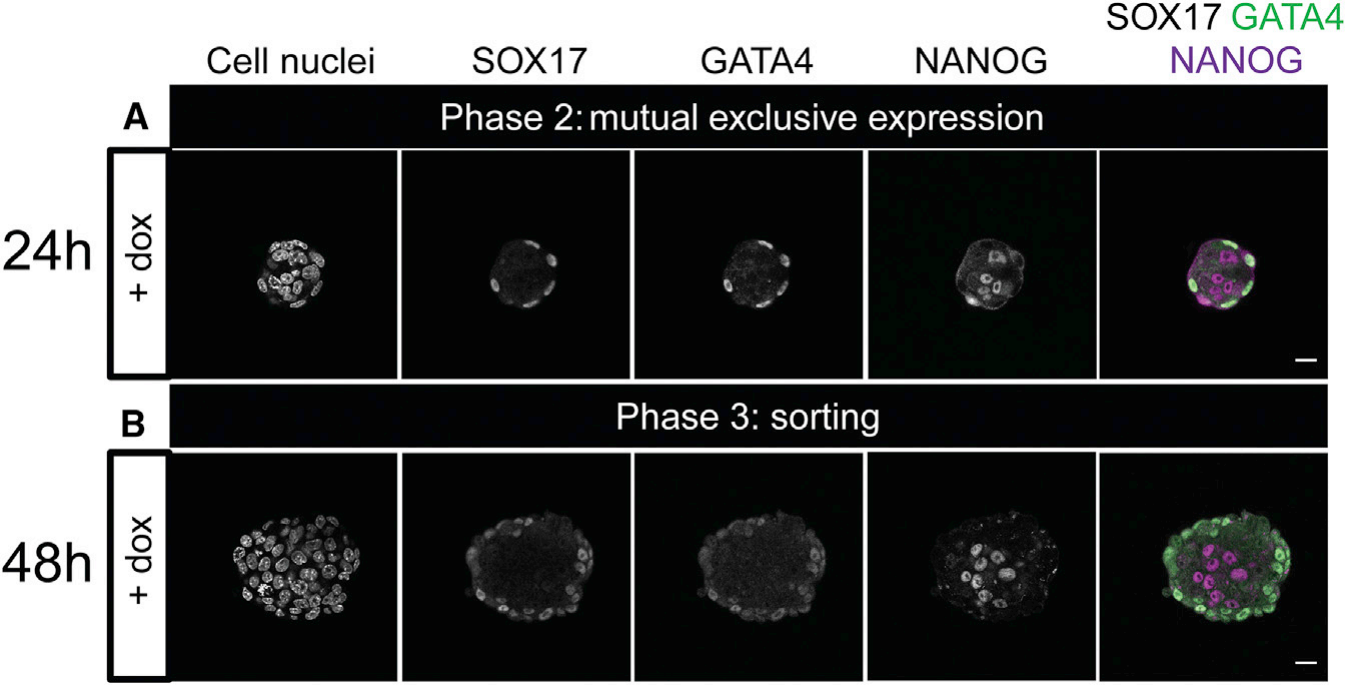
Mouse ICM organoids express the PrE markers GATA4 and SOX17. Mouse tet::GATA6 ESCs were stimulated for 6 h with doxycycline (+ dox) to induce PrE differentiation. After the removal of dox, 200 cells formed ICM organoids and were cultured for 24 h or 48 h. (*A*) GATA4 and SOX17 expression was found on the edge of the ICM organoids 24 h after formation. NANOG expression was found within the ICM organoids. (*B*) GATA4 and SOX17 expression was maintained 48 h after ICM organoid formation. Microscope: Zeiss LSM880; objective: 40×/1.3 oil differential interference contrast; scale bars, 20 *μ*m. To see this figure in color, go online.

Next, we checked for other PrE markers in the aggregates. Aggregates were created using a second cell line with a dox-inducible GATA6-FLAG transgene because GATA4 and GATA6 induction can be used interchangeably (16, 17). These ICM organoids were stained for SOX17 and GATA4, PrE markers that appear sequentially later in development (26). Both markers were expressed on the outer cell layer of the aggregate showing the same pattern as GATA6 expression (Fig. 3). The presence of GATA4 and SOX17 expression in these aggregates confirms the induction of the endogenous PrE program and the aggregate progression in development.

**Figure 4:**
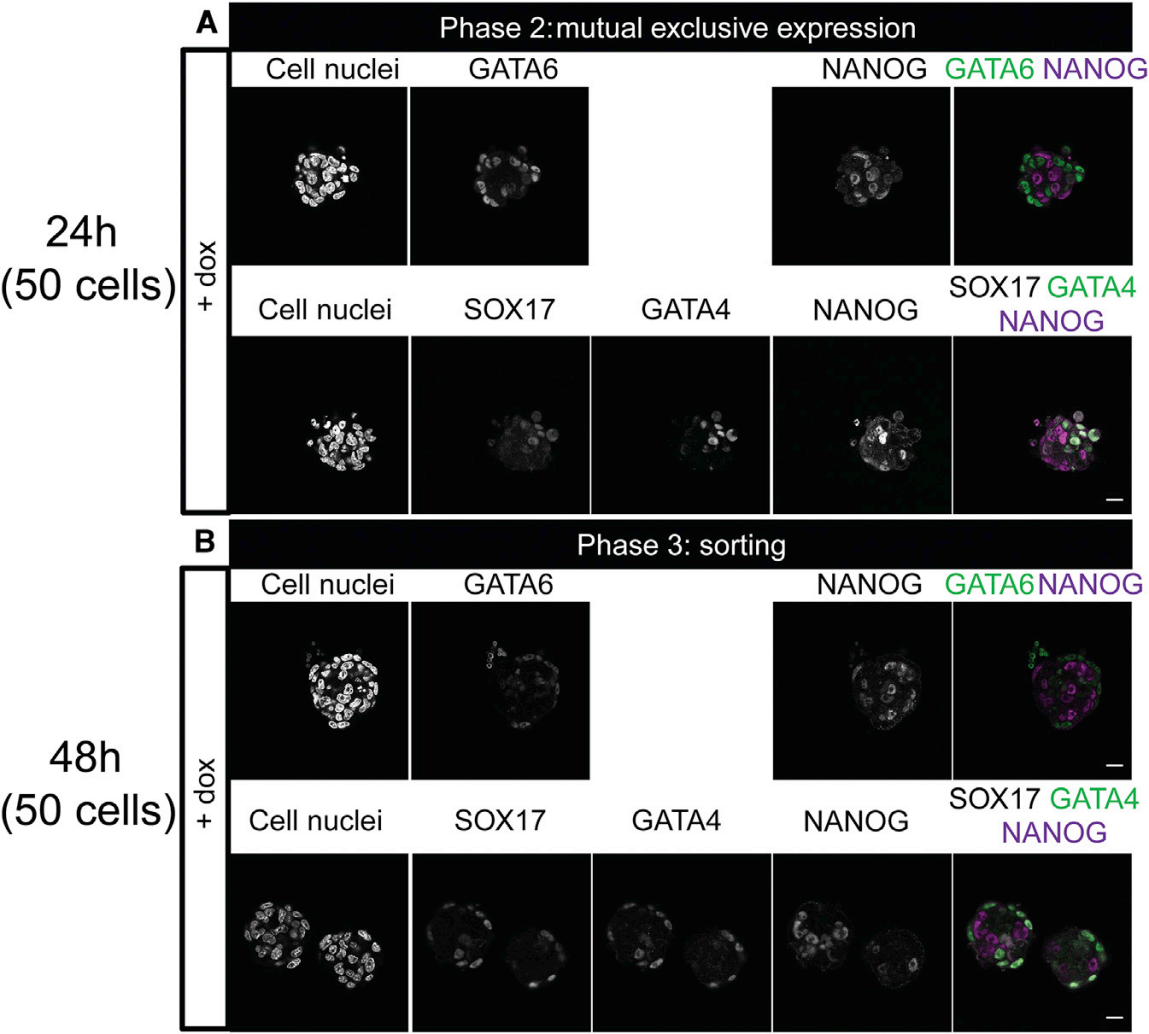
ICM organoids seeded with 50 cells show mutual exclusive expression and sorting. Mouse tet::GATA6 ESCs were stimulated for 6 h with doxycycline (+ dox) to induce PrE differentiation. ICM organoids were formed with 50 cells and kept undisturbed for 24 (*A*) and 48 h (*B*). ICM organoids show the two first phases: mutual exclusive expression and sorting of GATA6-positive cells. GATA4 and SOX17 expression was present at both stages. Microscope: Zeiss LSM880; objective: 40×/1.3 oil differential interference contrast; scale bars, 20 *μ*m. To see this figure in color, go online.

ICM organoids formed from tet::GATA6 cells appear smaller in size than the ones formed with tet::GATA4 (see Figs. 2 and 3). This difference arises from the differences in the protocol used for aggregate formation (for more details, see Materials and Methods). Despite the different cell lines and the variations in the formation process, the distinct phases were observed in both experimental setups. This underlines the robustness of our system. However, we wondered whether an even smaller cell number in the aggregates, closer to the mouse embryo size, influences their development. Therefore, we formed aggregates of 50 cells. We found the same two phases of mutual exclusive expression (Fig. 4). Furthermore, the PrE markers SOX17 and GATA4 show a similar pattern as in the aggregates of 200 cells. Hence, the cell number is not of primary importance for cell fate decision.

In summary, our data show that timing and differentiation of the ICM during preimplantation are mimicked by our ICM organoids even in the presence of LIF. Our results indicate that the cell number has no influence on cell fate decisions. Furthermore, because the ICM organoids contain no TE-like cells, a direct involvement of TE cells might not be required for PrE differentiation and sorting and the downregulation of NANOG in the Epi.

### ICM organoids show evidence of visceral and parietal endoderm formation

**Figure 5:**
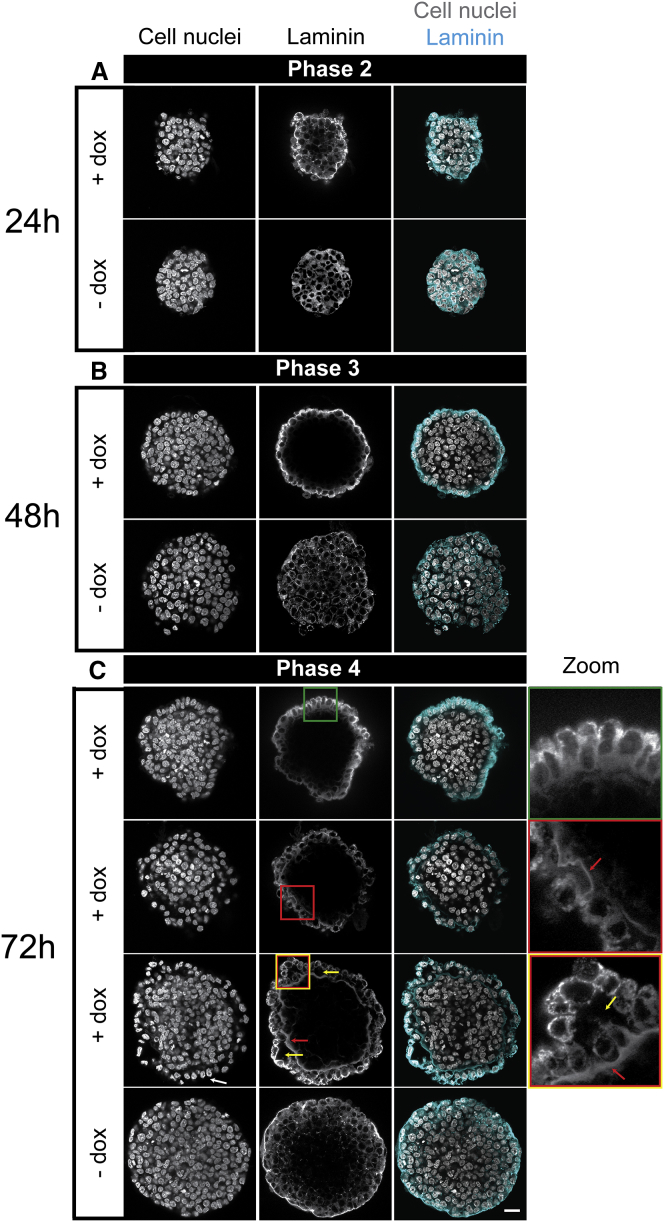
ICM organoids show secretion of basement membrane component laminin. Mouse tet::GATA4 ESCs were stimulated for 6 h with doxycycline (+ dox) to induce PrE differentiation. Cells that were not stimulated with dox (− dox) served as controls. (*A*) Heterogeneous laminin distribution in 1-day-old ICM organoids at stage of mutual exclusive expression. (*B*) After 48 h of ICM organoid formation, secretion of laminin is restricted to the outer layer. (*C*) After 72 h, at the stage of NANOG downregulation, laminin layers of different thicknesses were detected between the PrE layer and inner Epi core (*red arrows* and *red boxes*, *second* and *third rows*). Different structures could be observed and indicate differentiation toward visceral endoderm (VE) and parietal endoderm (PE): aligned columnar cell structure (*green box*, *first row*), aligned cuboidal cell morphology (*white arrows*, *third row*, *first column*), and vacuoles (*yellow arrows* and *box*, *third row*). Characteristics for VE were columnar- or cuboidal-shaped cells and low laminin expression; for PE, they were smaller-sized cells and loosely connected to the Epi core and high laminin expression. For more details, please also see text. Images show a single slice from the ICM organoids’ center. Microscope: Zeiss LSM780; objective: 63×/1.40 oil; scale bars, 20 *μ*m. To see this figure in color, go online.

In the embryo, a basement membrane consisting of extracellular matrix components, including laminin, assembles at the interface between Epi and PrE at peri-implantation stages (11). To investigate whether the developmental progression of the ICM organoids includes the presence and arrangement of a basement membrane, we performed anti-laminin staining at 24, 48, and 72 h after aggregation (Fig. 5).

One-day-old ICM organoids showed a bias for laminin enrichment at the edges, whereas the distribution in control aggregates was homogeneous (Fig. 5 *A*). After 48 h, outside cells in ICM organoids were clearly biased in their laminin localization, whereas in the controls, a homogeneous distribution throughout the aggregate was still visible (Fig. 5 *B*). After 72 h, the ICM organoids continued to show strong laminin expression in the outer cell layer. Remarkably, and in contrast to control aggregates, ICM organoids showed a ring of strong laminin staining between the outer PrE-like layer and the inner Epi-like core that also manifested as a physical gap between the two layers (see cell nuclei channel in Figs. 2 *D*, 5 *C*, and S3, 72 h). ICM organoids with a thick and prominent basement membrane even formed cavities on the outside (Fig. 5 *C*, *third row*, *yellow box* and *arrows*). This suggests that ICM organoids form a basement membrane between the Epi- and the PrE-like cell populations, similar to the situation in the embryo.

At 72 h, the control aggregates exhibit laminin mainly at the edge of the aggregate. This resembles similar structures previously seen in other types of three-dimensional cell cultures (27).

**Figure 6:**
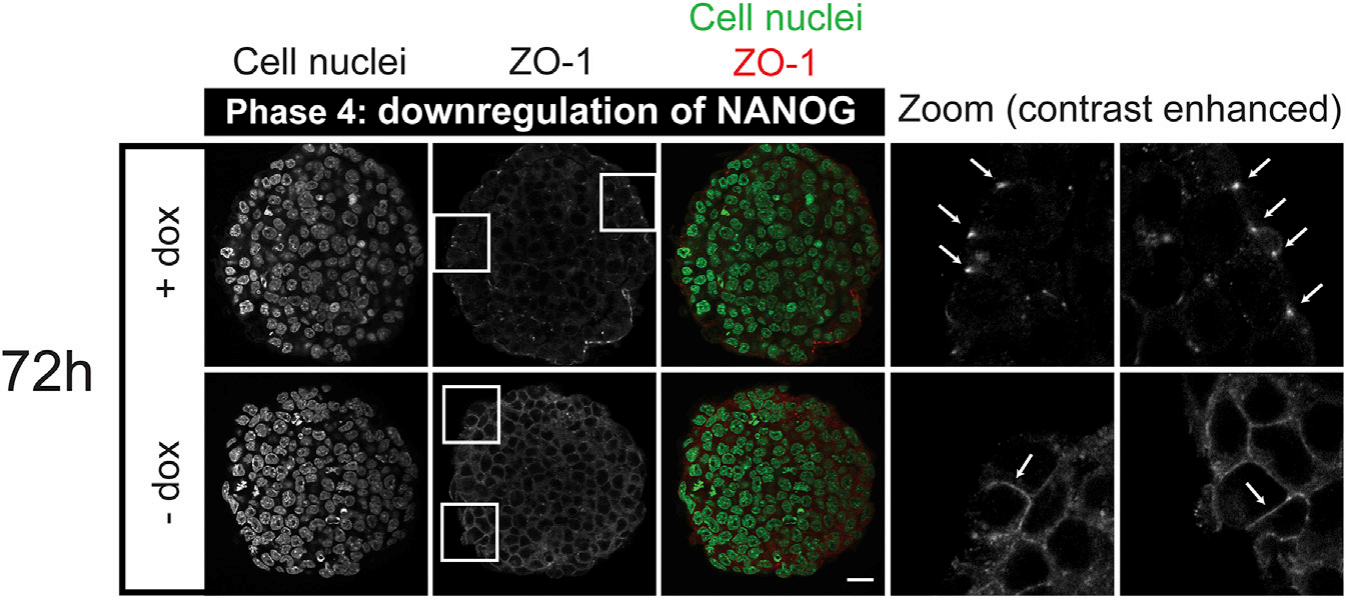
ICM organoids show characteristics of epithelisation. Mouse tet::GATA4 ESCs were stimulated for 6 h with doxycycline (+ dox) to induce PrE differentiation. Cells that were not stimulated with dox (− dox) served as controls. Aggregates were formed and kept undisturbed for 72 h. ICM organoids show punctate patterns of ZO-1 at the outer cell layer (see zoomed regions in *boxes* and *arrowheads*). Control aggregates show continuous ZO-1 staining at the junctions (see zoomed regions in *boxes* and *arrowheads*). Images show single slices from the ICM organoid’s center. Microscope: Zeiss LSM780; objective: 63×/1.40 oil; scale bars, 20 *μ*m. To see this figure in color, go online.

At 72 h, the ICM organoids also displayed a more irregular shape compared to the compact, round control aggregates (Figs. 2 *D*, 5 *C*, and S3, 72 h). The morphology of the cells in the outer layer differed strongly between ICM organoids and control aggregates. In the ICM organoids, we observed different types of structures. We found ICM organoids with cells in the outer layer that were accurately aligned and exhibited a columnar organization, reminiscent of a mature epithelium (Fig. 5 *C*, *first row*, *green box*). Staining with ZO-1, a marker for epithelialisation of the PrE that is exclusively found at tight junctions just beneath the apical surface of polarized epithelial cells (28), confirmed this idea: after 3 days, outer cells of the ICM organoids show a punctate ZO-1 pattern, whereas in the control aggregates, ZO-1 is expressed between cells (Fig. 6).

In further cases, the outer cells of ICM organoids were only loosely connected to the Epi-like cells with some detached completely, forming small cavities (Fig. 5 *C*, *second* and *third row*). In these cases, cell nuclei were cuboidal and, in some regions, aligned and oriented (Fig. 5 *C*, *first* and *third row*, *white arrow*).

The PrE differentiates into VE and PE. Previous studies show that VE and PE cells can be recognized by multiple characteristics. VE cells display a cuboidal or columnar morphology, whereas PE cells are smaller and not as tightly connected. VE cells express low levels of laminin, whereas PE cells express very high levels of laminin (13, 29). Altogether, the formation of basement membrane between PrE-like and Epi-like cells and the identification of VE and PE cell-specific characteristics in our ICM organoids suggest the differentiation of PrE-like cells toward VE-like and PE-like fates after 72 h.

### Lineage composition and spatial distribution of GATA6 and NANOG in ICM organoids and mouse embryos match for mid- and late mouse ICMs

The transcription factors GATA6 and NANOG are the key markers of PrE versus Epi specification (3, 4). Our visual inspection indicates strong similarities in the timing of fate decisions between ICM cells in blastocysts and in ICM organoids (Figs. 2, 3, 4, 5, and 6). Hence, we performed a quantitative spatial analysis of expression patterns of GATA6 and NANOG expressing cells in ICM organoids and mouse ICMs to evaluate the similarity in the three-dimensional organization of these two systems.

**Figure 7:**
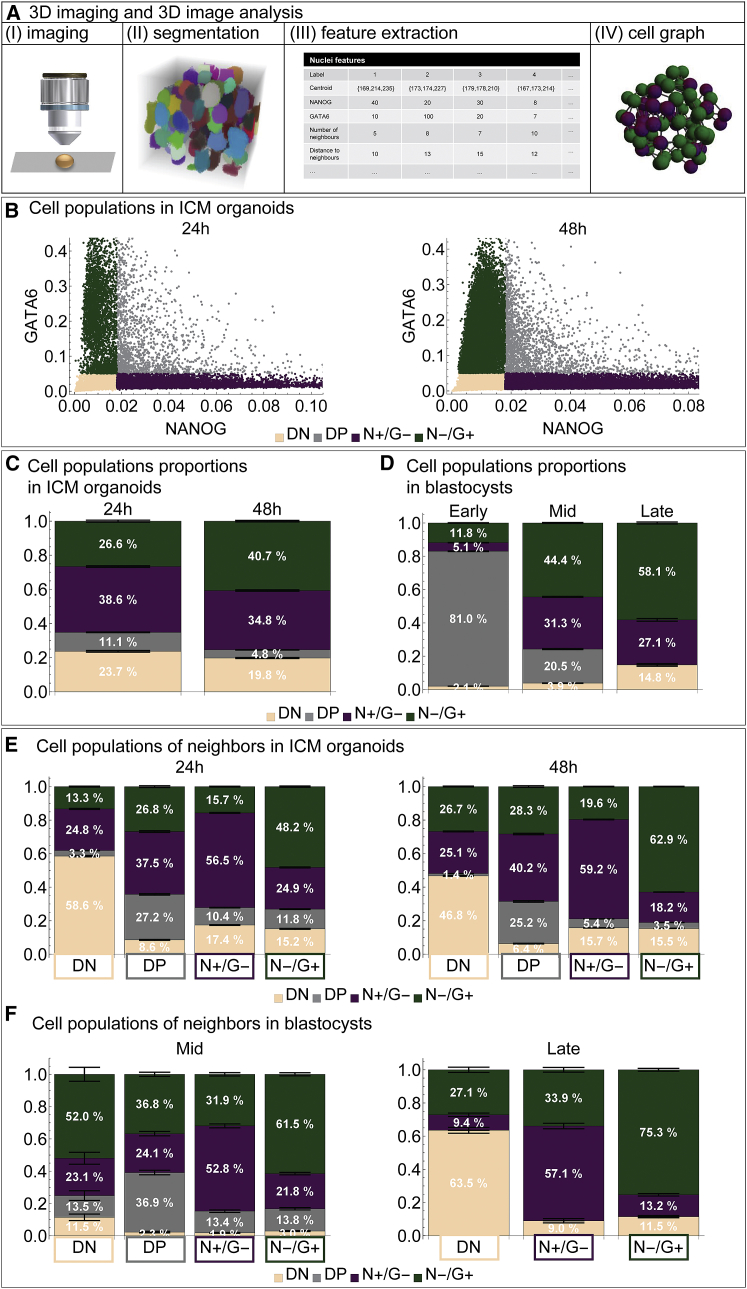
Lineage composition and spatial distribution of GATA6 and NANOG in ICM organoids resemble those of mid- and late mouse blastocysts. (*A*) Three-dimensional imaging and three-dimensional image analysis form the basis for a quantitative comparison between ICM organoids of mouse tet::GATA4 ES cells and blastocysts. Quantitative data of early, mid-, and late blastocysts were taken from Saiz et al. (22) (*B*) Fluorescence intensity levels of GATA6 and NANOG of individual cells in ICM organoids after 24 and 48 h. The data points are colored by cell population. (*C* and *D*) Lineage composition is shown as percentage of the total number of cells in ICM organoids after 24 and 48 h and in the ICM of early, mid-, and late blastocysts. (*E* and *F*) Lineage composition of neighboring cells is shown as percentage of the total of neighboring cells in ICM organoids after 24 and 48 h and the ICM of mid- and late blastocysts. The error bars indicate the standard error of the mean. The number of independent experiments for ICM organoids or blastocysts is, respectively, 76, 147. DN: double negative (NANOG−/GATA6−), DP: double positive (NANOG+/GATA6+), N+/G− (NANOG+/GATA6−), N−/G+ (NANOG−/GATA6+). To see this figure in color, go online.

We performed image-based quantitative single-cell analyses. The raw data consisted of three-dimensional images of fixed and immunolabeled ICM organoids (Fig. 7 *A*). We used our recently developed multiscale image analysis pipeline that combines automated cell nuclei segmentation with topological and graph analyses (18, 19). This pipeline provides more than 30 morphological features and fluorescent intensities of individual nuclei, the cellular neighborhood and the ICM organoid as a whole. Based on the intensity values, we used a previously proposed *k*-means clustering algorithm (22) to classify each cell as a member of one of the four populations: DN, DP, NANOG+/GATA6− (N+/G−, Epi precursor cells), and NANOG−/GATA6+ (N−/G+, PrE precursor cells) (Fig. 7 *B* and Materials and Methods). Analogous to (22), the data were log transformed, and the k-means clustering algorithm was used to determine the centers of three clusters—DN, N+/G−, and N−/G+. From these, the DP cells were determined, and the thresholds for NANOG and GATA6 were set as the minimal values of the respective expression values in the DP cells. We processed data for 76 ICM organoids, 34 of which were fixed and stained after 24 h and a further 42 after 48 h. ICM organoids were seeded with 200 cells, and after 24 h, they consisted of 442 ± 26 cells (Fig. S4 *A*). After 48 h, the cell number was significantly increased (*p* < 0.01) to 1041 ± 47 cells. Three-day-old ICM organoids were not included in the quantitative analyses because NANOG is downregulated at this stage (see Fig. 2 *D*).

We compared our data to available data from 147 mouse blastocysts (22). This data set contains lists of all relevant features for each nucleus in each embryo, similar to the feature list of our image analysis pipeline once features are extracted (Fig. 7 A III). We added the neighborhood features absent in the original embryo data set by calculating cell graphs (Fig. 7 A IV). In these three-dimensional cell graphs, pairs of vertices are connected by edges signifying a neighborhood relation between two cell nuclei. Because staging based on geometrical features such as cell number is not appropriate for our comparative analysis, we grouped the blastocysts into three developmental stages based on the progress of their development (19). Because the proportions of the four cell populations are a strong indicator for the development of mouse embryos, we used this criterion to stage the embryos (22, 30). Early blastocysts correspond to the stage at which GATA6 and NANOG are coexpressed in each cell, and hence, more than 55% of ICM cells are DP (70 embryos). Mid-blastocysts show a mutually exclusive expression with less than 55% DP cells (53 embryos). Embryos with no DP cells were assigned to the late blastocyst stage (24 embryos). Early blastocyst ICMs consisted of 21 ± 1 cells and mid-blastocyst ICMs of 29 ± 1 cells, and the number increased to 44 ± 4 cells in late blastocysts (Fig. S4 *B*).

In ICM organoids, the proportion of DN and N+/G− cells remained constant within 24 h (*p* > 0.05, 0 in confidence interval (CI)) (Fig. 7 *C*; Table S2). The proportion of DP cells decreased, and the proportion of N−/G+ cells increased significantly (*p* < 0.01). This indicates that the cell fate specification is progressing from 1- to 2-day-old ICM organoids. To assess whether this progression in cell fate specification is comparable to the development in mouse embryos, we determined cell numbers for the four cell populations in the ICMs (DN, DP, N+/G−, N−/G+) (Fig. S5) (22). As previously described, the proportion of DP cells decreases dramatically from early to mid-blastocysts (*p* < 0.01). The proportion of N+/G− cells increased mainly from early to mid-blastocysts (*p* < 0.01), whereas the proportion of N−/G+ cells increased all throughout blastocyst development (*p* < 0.01). These numbers illustrate the shift from coexpression to a mutually exclusive expression of GATA6 and NANOG in the ICM of mouse blastocysts.

We next compared the lineage composition of ICM organoids (Fig. 7 *C*) with the corresponding results for the ICM of mouse blastocysts (Fig. 7 *D*; Table S3). The proportion N+/G− cells in ICM organoids after 24 h is comparable to the proportion of this population in mid-blastocysts (*p* > 0.05, 0 in CI). Furthermore, the proportions of DN cells and N+/G− cells were comparable between 48 h and late blastocysts (*p* > 0.05, 0 in CI). The proportion of Epi precursor cells (N+/G−) did not change between 24 and 48 h in ICM organoids (*p* > 0.05). This finding agrees well with the constant number of Epi precursor cells in mid- and late blastocysts (*p* > 0.05). The relative increase of PrE precursor cells (N−/G+) is also similar between ICM organoids and mid- to late blastocysts. Together, a decrease in the number of coexpressing cells, an increase in the number of PrE cells and no changes in the number of Epi cells were observed in the transitions from 24 to 48 h ICM organoids as well as from mid- to late blastocysts. The proportion of DN cells started at a higher level in 24 h ICM organoids compared to mid-blastocysts and stayed at a similar level in 48 h ICM organoids that was comparable to late blastocysts. No correlation was observed between the number of DN cells and the aggregate size.

Finally, we characterized the spatial distribution of the cells. We measured the neighborhood composition of each cell population in both systems using the calculated cell graphs (Fig. 7, *E* and *F*). DN cells in ICM organoids (24 and 48 h) were mostly surrounded by DN cells (*p* < 0.01). In the mid-blastocyst, mostly GATA6 cells accumulated around DN cells (*p* < 0.01), and in the late blastocyst, it shifted toward DN cells (*p* < 0.01). In ICM organoids, DP cells had a similar proportion of DP and N−/G+ neighbors after 24 h (*p* > 0.05). In the mid-blastocyst, more N−/G+ cells were found in the neighborhood of DP cells (*p* < 0.01). Interestingly, the neighbor composition of N+/G− and N−/G+ cells showed a striking similarity between the 24 h ICM organoids and mid-blastocysts or 48 h ICM organoids and late blastocysts, respectively. In both systems, Epi precursor cells are mostly surrounded by cells with the same identity, and PrE precursor cells have mostly neighbors that are PrE precursor cells. Furthermore, for several lineages in the neighborhood, the ratio is comparable between 24 h ICM organoids and mid-blastocysts, as well as 48 h ICM organoids and late blastocysts (Table S4, marked in *gray*).

In summary, these data demonstrate that both the ratios of different cell types as well as their spatial arrangement are in broad agreement between 1-day-old ICM organoids and mid-blastocysts as well as 2-day-old ICM organoids and late blastocysts, even though the number of cells in the two systems differed by a factor of 10. In particular, we find in both systems and at all stages that Epi and PrE precursor cells are mainly surrounded by cells of the same population.

### ICM organoids show local clustering of cells with the same fate

Cells of the ICM shift from coexpression to mutually exclusive expression of GATA6 and NANOG during embryonic development. The distribution of NANOG and GATA6 at this stage is often referred to as a salt-and-pepper pattern. Although no definitive definition of this type of pattern exists, it is usually thought to indicate a random intermingling of Epi and PrE cells in the ICM (12, 31). This idea contrasts with several recent theoretical studies that model the influence of Epi cells on neighboring cells to induce PrE cell fate via a short-range FGF signal (6, 7, 8, 32) and that are therefore suggestive of an alternating patterning between neighboring cells. We reasoned that a careful classification of the spatial patterns of cell fates in the ICM organoid would put those models to the test and provide an experimental constraint on the activity range of the signals that mediate communication between the Epi and the PrE.

**Figure 8:**
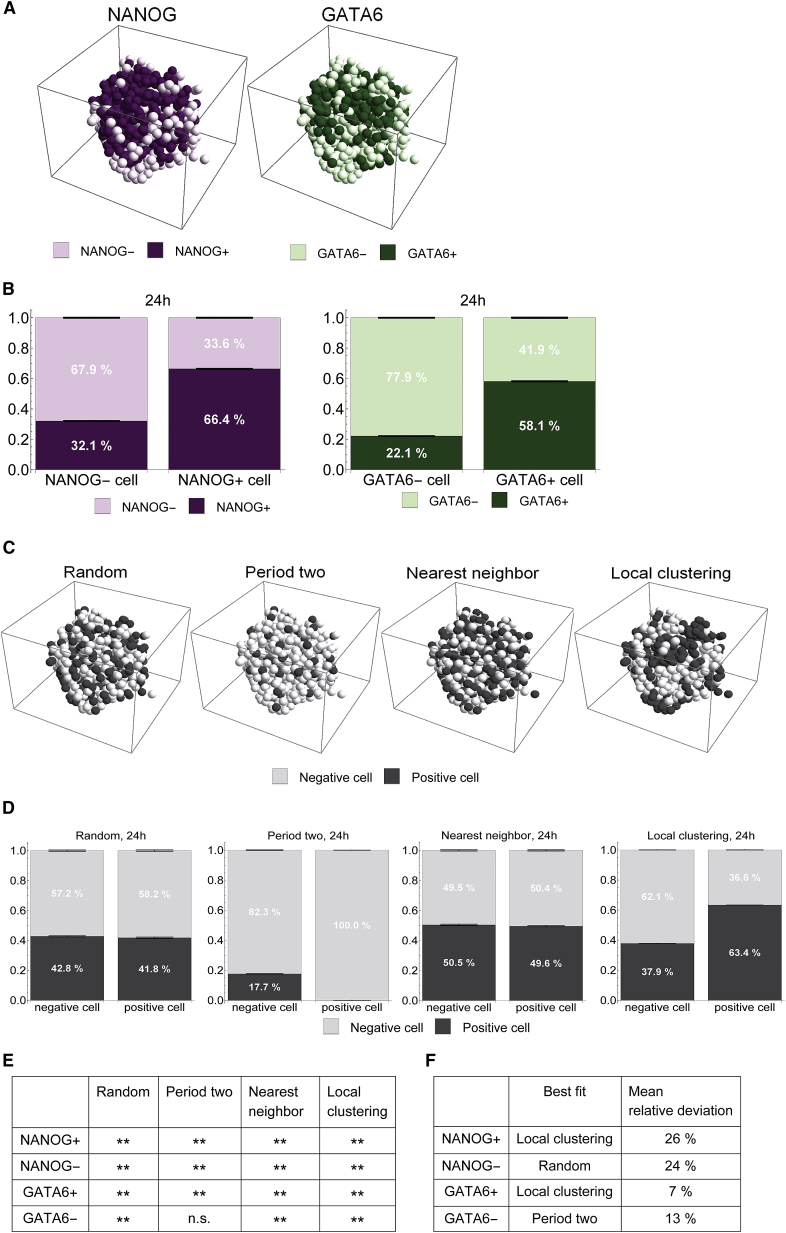
Mutual exclusive pattern in 1-day-old ICM organoids exhibits local cell fate clustering. (*A*) Spatial distribution of NANOG-positive and negative cells or GATA6-positive and negative cells, respectively, in a 1-day-old ICM organoid (positive: N+/G− or G+/N−, respectively, and DP; negative: N−/G+ or G−/N+, respectively and DN) (*B*) Lineage composition of neighboring cells is shown as percentage of the total number of neighboring cells in ICM organoids after 24 h. The error bars indicate the standard error of the mean. (*C*) Simulation of three artificial patterns describing the salt-and-pepper patterning (for more information, please see Materials and Methods). A negative cell is NANOG-negative or GATA6-negative, respectively. A positive cell is NANOG-positive or GATA6-positive, respectively. (*D*) Lineage composition of neighboring cells is shown as percentage of the total of neighboring cells in the three simulated artificial patterns. The error bars indicate the standard error of the mean. (*E*) Statistical comparison of the data from 24-h-old ICM organoids with the three artificial patterns (^∗∗^*p* < 0.01, n.s.: not significant; for details, Wilcoxon-Mann-Whitney test with Bonferroni correction). (*F*) The closest fit of the simulated pattern to the experimental data is expressed as mean relative deviation, i.e., $\left(\bar{s}-\bar{d}\right)/\bar{d}$, where $\bar{s}$ is the mean of the simulated data and $\bar{d}$ the mean of the experimental data. Number of independent experiments for ICM organoids: 34. To see this figure in color, go online.

To investigate the distribution of cell fates in 1-day-old ICM organoids, we divided them into NANOG-negative and NANOG-positive cells or GATA6-negative and GATA6-positive cells, respectively (Fig. 8 *A*). The division was based on the previously determined cell populations. Hence, DN and N−/G+ cells were combined to NANOG-negative cells, DP and N+/G− cells were combined to NANOG-positive cells, and the definitions for GATA6-negative and positive cells are analogous. The analyses of neighborhood compositions reinforce the previous result that cells are surrounded by cells with the same fate (Fig. 8 *B*).

Next, we compared the distribution in ICM organoids with four rule-based simulated patterns. The purpose of the simulations is to determine whether the complex three-dimensional patterns can be broken down to simple rules. The first three describe possible forms of the salt-and-pepper pattern, and the fourth pattern is a local cell fate clustering (Fig. 8 *C*): 1) random pattern: expression levels were randomly assigned to the cells; 2) period two pattern: positive cells are solely surrounded by negative cells (23); 3) nearest-neighbor pattern: one cell was assigned a negative state. Its nearest neighbor was assigned the opposite state, thus positive. This was iteratively continued until all cells were assigned a state; 4) local clustering: one cell was assigned a state (negative or positive). Its nearest neighbor was assigned the same state with 90% probability, a value determined by a parameter scan (see Materials and Methods for further details). This was continued until each cell was assigned a state. The neighborhood composition was analyzed for all four simulated patterns (Fig. 8 *D*). Comparison between the pattern found in 24 h ICM organoids and the simulated patterns revealed that the experimentally observed patterns for positive and negative cells together were significantly different from all simulated patterns (Fig. 8 *E*). Solely for GATA6-negative cells, the neighborhood composition was comparable to that of negative cells in the period two pattern. To determine the simulated patterns that were closest to the patterns for the other three cell types individually, we used the effect sizes, i.e., the relative difference between the simulations and the experimental data (Fig. 8 *F*). The neighborhood distribution of NANOG-positive cells and GATA6-positive cells were best resembled by the local clustering pattern. For NANOG-negative cells, the neighborhood distribution was closest to the random pattern, whereas the distribution of GATA6-negative cells was best approximated by a period two pattern.

Next, we evaluated the potential of the three best matching patterns for the two cell states (positive and negative) in the context of four populations (DN, DP, N+/G−, N−/G+). We generated four simulations that each combined two of the best matching patterns (Fig. S6). We find that none of the simulations for four populations can adequately reproduce the clustering in N+/G− and N−/G+ cells. Overall, a random pattern for NANOG and a period two pattern for GATA6 provide the best match of the simulations to the experimental data. Simulations for 2-day-old ICM organoids were not performed because at this stage, sorting has almost finished.

Our data provide details of the expression pattern during PrE-like differentiation at a time point between the onset, at which all cells are DP, and the final stage, at which the two cell types are clearly sorted. Even though the pattern cannot comprehensively be described by simple rules, the results strongly indicate that the distribution of Epi-like and PrE-like cells in 24 h ICM organoids does not generally follow a salt-and-pepper pattern, in which Epi-like and PrE-like cells intermingle randomly, but rather shows local clustering of cells of the same fate.

## Discussion

In this study, we present a, to our knowledge, novel three-dimensional stem cell system termed “ICM organoids” based on mESCs that are inducible to PrE-like differentiation. Our ICM organoids show key events and timing of cell fate specification in the ICM of mouse blastocysts. Applying our three-dimensional quantitative neighborhood analyses revealed that contrary to previous assumptions, GATA6- and NANOG-expressing cells show local clustering both in ICM organoids and mouse embryos. Our results for the spatial patterns of cell fates put existing models to the test and provide constraints for the cellular mechanisms governing Epi versus PrE differentiation. We expect ICM organoids to become a useful tool for studying the molecular mechanisms underlying Epi versus PrE differentiation in mammals.

### ICM organoids resemble events and timing of mouse ICMs during the preimplantation phase

EBs form an outside layer of cells with endodermal characteristics, which have been interpreted to mirror the PrE of the preimplantation embryo (14, 33). However, a continuous outer endodermal layer is usually only observed at day 4 after aggregation in EBs, which is significantly longer than PrE formation in the embryo. Given that most molecular markers cannot distinguish between the PrE and the definitive endoderm, this leaves the possibility that the endodermal layer in EBs may be composed mostly of cells with definitive rather than PrE characteristics.

Here, we use induced expression of GATA factors to differentiate ESCs into a cell type with a proteome that resembles embryo-derived XEN cells, a tissue culture model of the PrE (17, 34). Using this cell system to form three-dimensional aggregates, we detect a continuous layer of cells with PrE characteristics as early as 2 days after aggregation. This temporal difference in the formation of the endodermal layer between the ICM organoids and EBs indicates that our system approximates cell fate decisions in the ICM better than previous in vitro approaches.

The mutually exclusive expression pattern of Epi and PrE markers emerges in the ICM from ∼E3.0 to E3.75 (2). This schedule is reproduced in our ICM organoids: the earliest occurrence of the mutually exclusive pattern was observed after 18 h (data not shown). From ∼E3.75 to E4.5, cell sorting is finalized in blastocysts (2). This is similar in our ICM organoids that complete sorting within 1 day. The distribution of cell types as well as neighborhood configuration during these two phases is in good agreement between the mouse ICM and the ICM organoids. In the late blastocysts, NANOG expression is rapidly downregulated from E4.5 onwards (10). In our ICM organoids, NANOG downregulation is initiated after 2 days. After 3 days, laminin formed a layer of varying thickness in ICM organoids. Before implantation, the Epi of mammalian embryos is separated from a layer of PrE by a basement membrane (11). During implantation, PrE cells further differentiate into PE and VE. Some of the 3-day-old ICM organoids also show a progress in PrE development. The configuration of the basement membrane and the PE-like cells seems to depend on the size of the ICM organoid. We hypothesize that first the PrE cells differentiate into PE cells, and then the PE cells start to form a layer that detaches from the ICM organoid, resulting in the small cavities. Extending the cultivation time for the ICM organoids combined with time-lapse imaging of the development will help to test this hypothesis.

In later stages of the ICM organoids, the increased occurrence of DN cells further illustrates the lineage maturation of Epi and PrE cells. This is reflected by the observed downregulation of NANOG and the identification of PrE markers (SOX17 and GATA4) in the ICM organoids, which appear sequentially later in development (26).

Seeding cells into multiwell plates with a concave, nonadhesive surface provides a robust way of generating aggregates with a variation in cell number that resembles that of mouse embryos. The number of cells in ICM organoids approximately doubled within 1 day. Live-cell imaging and tracking of the ICM cells have shown that cells divide at least once during 20–30 h of imaging (35). Thus, cell-cycle progression in ICM organoids is also comparable to that in the in vivo system.

**Figure 9:**
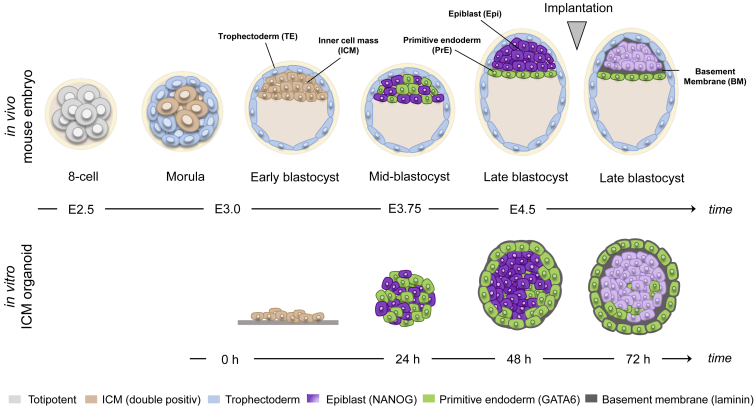
In vitro ICM organoids closely resemble the ICM of in vivo blastocyst stages during mouse preimplantation. A schematic representation of ICM organoid development relative to the different stages in the preimplantation mouse embryo. To see this figure in color, go online.

Together, our results demonstrate that key events and timing in ICM organoids closely resemble those of cell fate specification in mouse ICMs despite the lack of a TE and the large difference in cell number (Fig. 9).

### The TE provides a mechanical constraint

Only little evidence has been provided that TE cells affect ICM cell fate decision. It is indicated to be functionally relevant but not deterministic in regard to guiding the ultimate fate (36). The subordinate role of the TE is further underlined by the fact that ICM organoids that lack TE cells can resemble important cell fate decision stages of ICM cells of mouse blastocysts. We hypothesize that a major function of the TE is to serve as a mechanical constraint. The PrE-like cells in the 2- and 3-day-old ICM organoids enclose the cell aggregate. In the mouse embryo, PrE cells form a cup at only one end of the Epi. Simulations have shown that a chemical or a mechanical bias is required to establish such an asymmetric distribution of the PrE cells (37). This hypothesis can be tested in future studies by adapting the ICM organoid environment in our system either mechanically or chemically.

### Interaction of GATA6- and NANOG-expressing cells is important for Epi maturation

For the generation of ICM organoids, we optimized several steps in the protocol. In previous studies, induction of PrE differentiation has been conducted solely by mESC aggregation in the absence or presence of LIF from the culture medium (13, 14). We induced directed differentiation by a dox-induced expression of GATA factors followed by three-dimensional aggregation in the presence of LIF. This led to an interesting NANOG downregulation in later stages, even though LIF is still present in the medium. Because we saw no increase in apoptosis in the center of these ICM organoids, we can rule out that the basement membrane acts as a physical barrier for relevant factors. The downregulation of NANOG is reminiscent of that in the embryo. Little is known about its initiation. Two hypotheses are discussed in Artus et al.: either inhibition is induced by FGF4 expression or indirectly through PrE differentiation (10). In ICM organoids, NANOG downregulation only occurred in conjunction with PrE differentiation. Therefore, our data suggest the existence of signaling events between GATA6-positive cells and Epi-like cells in our system that lead to Epi and PrE maturation.

### Distribution of PrE and Epi fate exhibits local clustering

Early stages of PrE differentiation (1-day-old ICM organoids and mid-blastocysts) are characterized by a complex three-dimensional spatial arrangement of cells with opposite fates. We found that patterning of NANOG and GATA6 at the stage of 24 h ICM organoids appears in clusters of identical fates. Our data for the neighborhood composition in mouse blastocysts together with a previous study (19) indicate that cell fate clustering is also present in vivo.

It is likely that the three-dimensional arrangement of cell fates reflects the mechanisms that drive fate choice from the NANOG/GATA coexpression state. It remains unclear at what point the earliest pattern arises. In mouse embryos, we observed distinct spatial expression patterns already in early embryos (19).

The 24-h-stage ICM organoids are comparable to mid-stage embryos. For mid-stage embryos, there is a conflict between two models that favor either a mechanism based on intercellular signaling (38, 39, 40) or a cell division mechanism (41). The latter involves multiple waves of asymmetrical divisions that drive the patterning. The signaling mechanism has been proposed to depend on FGF. In this model, a non-cell-autonomous FGF signal emanating from an Epi cell would favor the PrE fate in the neighboring cell (6, 7, 8, 32). This would result in cells of opposite fates neighboring each other, which does not agree with our data. If fate clustering were mediated by FGF4, this would suggest the existence of local signaling hotspots that might be fueled by a community of Epi cells and promote the PrE fate in groups of cells. Investigating FGF/ERK signaling in ICM organoids directly, as well as measuring the spatial range of the FGF signal emanating from single cells, will be crucial to test this. Alternatively, it is possible that local clustering of cell fates is a consequence of fate decisions in combination with cell divisions that are likely to result in sister cells with the same fate. Lastly, local clustering might also arise from a convolution of the molecular processes that establish discrete fates with subsequent sorting events.

Time-lapse imaging as well as comparison of simulation results from existing mathematical models with our data will provide further insight.

## Conclusions

ICM organoids serve as an effective complementary approach to mouse embryos for studying cell fate specification during preimplantation. Because of their minimalistic nature, they allow focusing on the role of the ICM and enable approaches that are challenging to perform in vivo. Establishing the similarities, but even more so, the differences between ICM organoids and mouse embryos will further our understanding of the key components that drive PrE differentiation. Recently developed mathematical models suggest different kinds of mechanisms for cell fate specification (6, 7, 16). The three-dimensional data from our in vitro system provide a starting point to test mathematical-physical, i.e., quantitative, models.

An extension of our approach to heterotypic ICM organoids allows a detailed investigation of local cell interactions. The combination of ICM organoids with new reporters and live-cell imaging extends the possibilities for gaining more detailed insights into lineage specification. In the future, our neighborhood analyses can also be applied to other in vivo systems or ICM organoids derived from further mammalian organisms such as rat, pig, or even human stem cells. This provides a powerful approach for quantitative studies of embryogenesis.

The data sets used and/or analyzed during this study are available from the corresponding author on reasonable request.

## Author Contributions

Conception and design of the study were performed by B.M. and S.C.F.; B.M., E.H.K.S., and S.C.F. developed the methodologies. Data were acquired by B.M. and E.C.-S.; S.C.F. analyzed the data. B.M., S.C.F., S.M.-D., E.C.-S., E.H.K.S., and C.S. interpreted the data. B.M. wrote the manuscript. All authors revised the manuscript. S.C.F. supervised the study.

## Supporting Material

Document S1. Figs. S1–S6 and Tables S1–S4

Document S2. Article plus Supporting Material
