# Supplementary material for: Mouse ICM Organoids Reveal Three-Dimensional Cell Fate Clustering
Source: Biophys J. 2018 Nov 16;116(1):127–41. doi: 10.1016/j.bpj.2018.11.011 (PMC6341222; doi:10.1016/j.bpj.2018.11.011)
Supplement: Document S1. Figs. S1–S6 and Tables S1–S4 [file mmc1.pdf]

Biophysical Journal, Volume *116*

Supplemental Information

Mouse ICM Organoids Reveal Three-Dimensional Cell Fate Clustering

Biena Mathew, Silvia Muñoz-Descalzo, Elena Corujo-Simon, Christian Schröter, Ernst H.K. Stelzer, and Sabine C. Fischer

Supplementary figures and tables

Mouse ICM organoids reveal three-dimensional cell fate clustering

Authors

Biena Mathew[1], Silvia Muñoz-Descalzo[2,3], Elena Corujo-Simon[3], Christian Schröter[4], Ernst H. K. Stelzer[1], Sabine C. Fischer[1,*]

[1]Physikalische Biologie, Fachbereich Biowissenschaften, Buchmann Institute for Molecular Life Sciences, Goethe-Universität Frankfurt am Main, Frankfurt am Main, Germany; [2]Department of Biology and Biochemistry, University of Bath, Bath, United Kingdom; [3]Instituto Universitario de Investigaciones Biomédicas y Sanitarias, Universidad Las Palmas de Gran Canaria, Las Palmas de Gran Canaria, Spain; and [4]Department of Systemic Cell Biology, Max-Planck-Institute of Molecular Physiology, Dortmund, Germany

*Correspondence: sabine.fischer@uni-wuerzburg.de

Elena Corujo-Simon's present address is Wellcome Trust-Medical Research Council Cambridge Stem Cell Institute, University of Cambridge, Cambridge, United Kingdom.

Sabine C. Fischer's present address is Center for Computational and Theoretical Biology, Faculty of Biology, Universität Würzburg, Würzburg, Germany

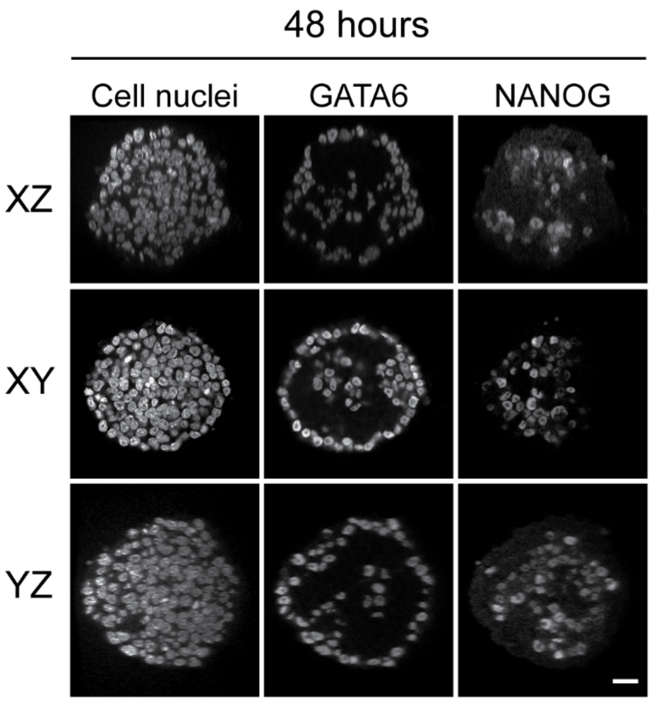

Figure S1. Views along xz, xy and yz of an ICM organoid formed for 48 hours. Images show single slices from the centre of a two-day old ICM organoid for each channel and along indicated directions. Microscope: Zeiss LSM780, objective: 63x/1.40 oil, scale bar: 20 µm.

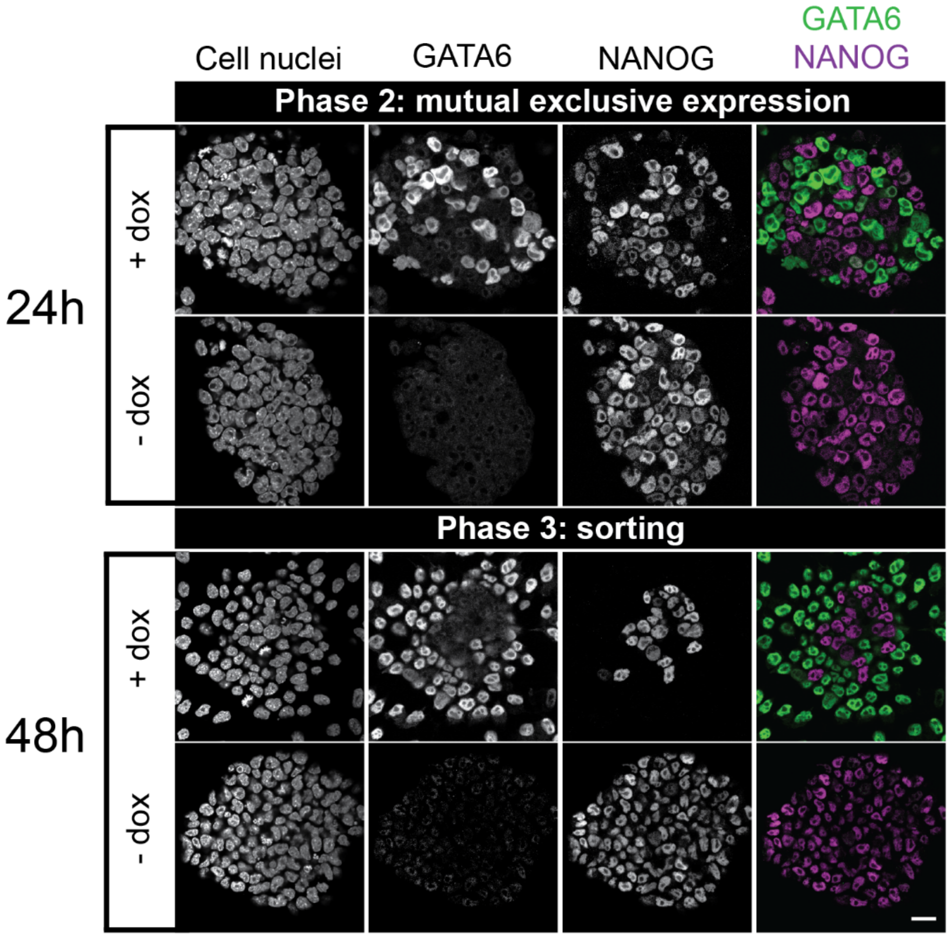

Figure S2. Mouse embryonic stem cells (mESC) show mutual exclusive expression and cell sorting. Mouse tet:GATA4 ES cells were stimulated for six hours with doxycycline (+ dox) to induce primitive endoderm differentiation. Cells that were not stimulated with dox (-dox) served as control. (A) 24 hours after dox removal, GATA6 and NANOG were mutually expressed within the colonies. (B) After 48 hours, GATA6-positive cells occupied the rim of the colonies. Microscope: Zeiss LSM780, objective: 63x/1.40 oil, scale bar: 20 µm.

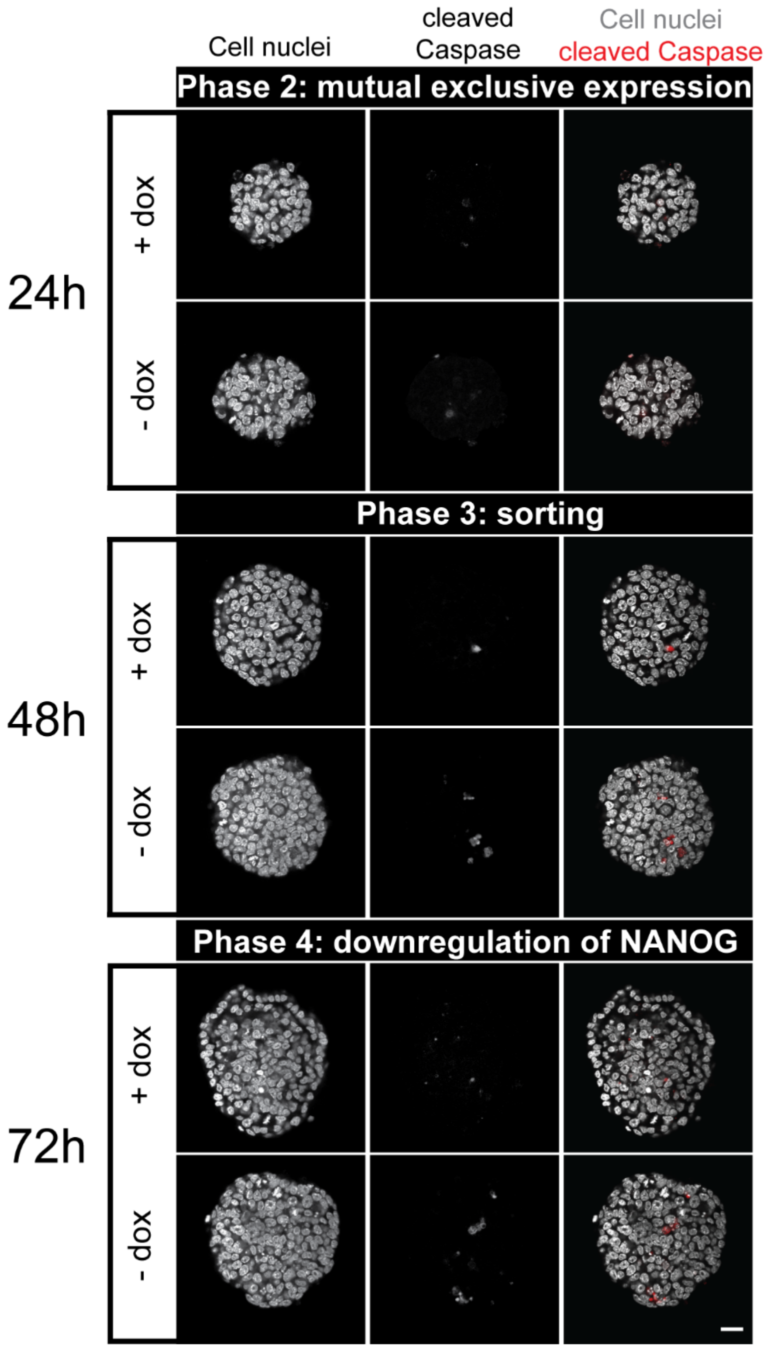

Figure S3. ICM organoids did not show increased apoptosis during experiment. Mouse tet:GATA4 ES cells were stimulated for six hours with doxycycline (+ dox) to induce primitive endoderm differentiation. Cells that were not stimulated with dox (- dox) served as control. Aggregates were formed and kept undisturbed for (A) 24, (B) 48 and (C) 72 hours. Activity of cleaved caspase (red) was not markedly increased during cultivation. Images show a single slice from the ICM organoid centre. Microscope: Zeiss LSM780, objective: 63x/1.40 oil, scale bar: 20 µm.

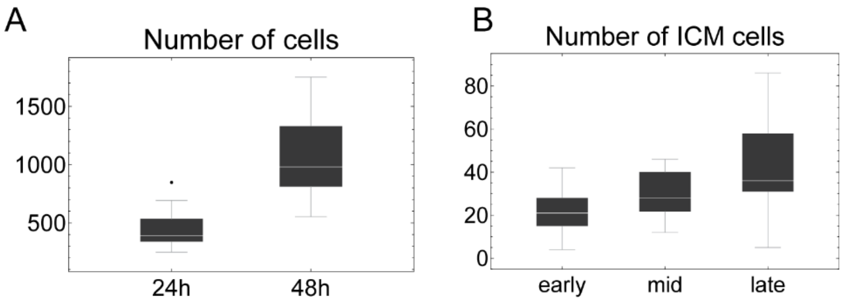

Figure S4. Number of cells in ICM organoids and the ICM of blastocysts. (A) Number of cells in ICM organoids after 24 hours and 48 hours and (B) the number of cells in the ICM for early, mid and late blastocysts. The error bars indicate the standard error of the mean. Number of independent experiments for ICM organoids or blastocysts, respectively: 76, 147.

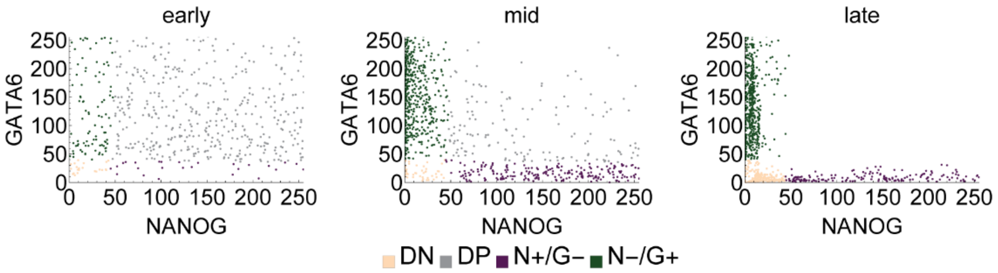

Figure S5. Fluorescence intensity levels of NANOG and GATA6 of individual ICM cells in early, mid and late blastocysts. The data points are colored by cell population. DN: double negative (NANOG-/GATA6-), DP: double positive (NANOG+/GATA6+), N+/G- (NANOG+/GATA6-), N-/G+ (NANOG-/GATA6+). Number of independent experiments for blastocysts: 147.

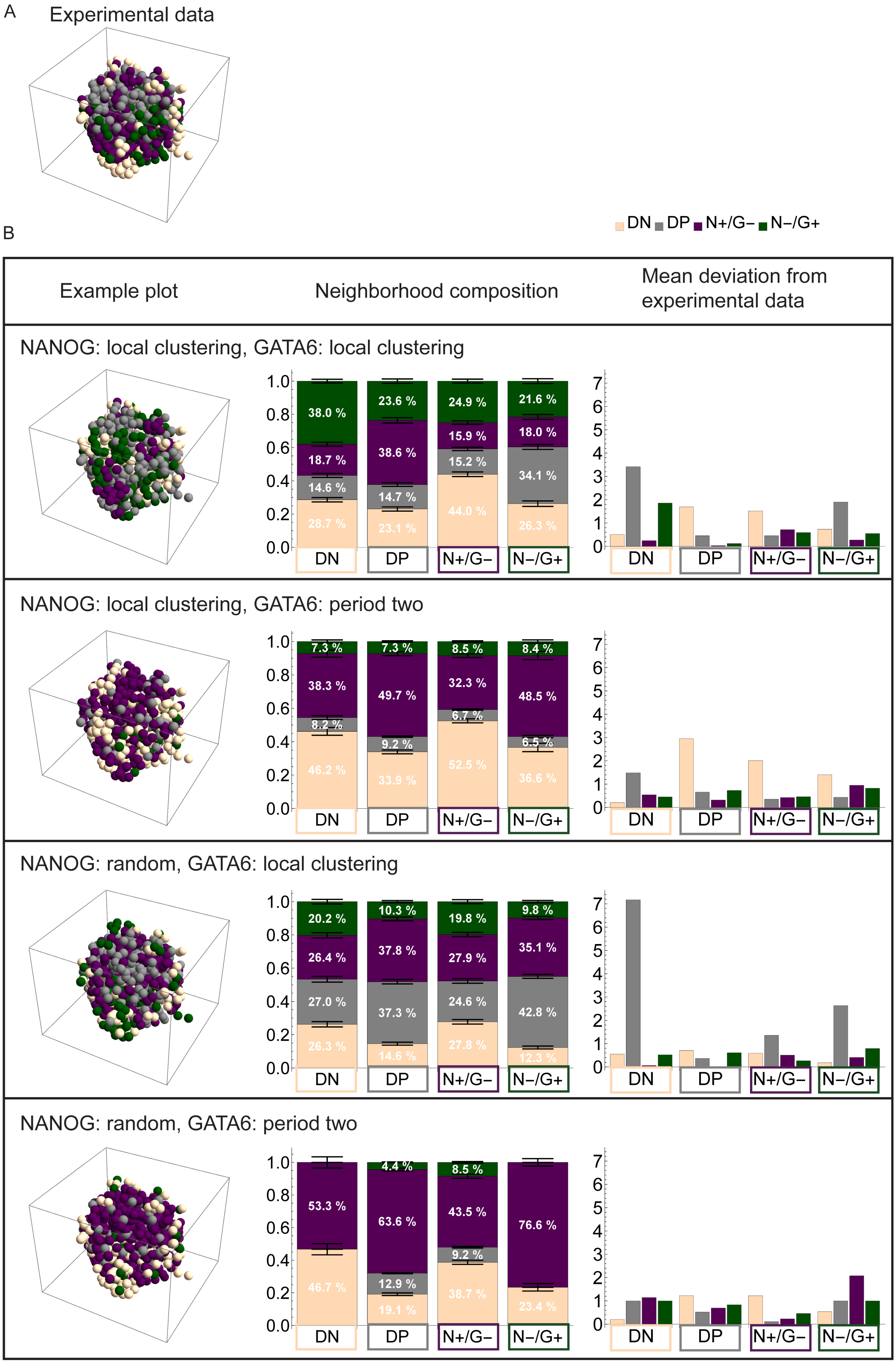

Figure S6. The combination of random pattern for NANOG and period two for GATA6 provides the closest match with experimental data. (A) Spatial distribution of the four populations in a one-day old ICM organoid. (B) Example plots, neighborhood composition and mean deviation from experimental data for four simulated patterns for four populations (further details are contained in the method section).

Table S1 Parameter names and values used for cell nuclei segmentation, alpha shape and cell graphs of the ICM organoids.

**Segmentation**	
**Parameter**	**Value**
ImageZScalingFactor	8
ImageScalingFactor	0.5
NucleiSeedDetectionMinRadius	9
NucleiSeedDetectionMaxRadius	12
NucleiFilterRange	3
NucleiThresholdRange	22
NucleiMeanFactor	0.75
NucleiSeedDilation	2
NucleiBackgroundFactor	0.35
NucleiMinCount	10
NucleiMaxCount	45,000

**Alpha shape / cell graphs**	
**Parameter**	**Value**
Alpha	90
OutlierDistanceThreshold	30
EdgeDistanceThreshold	91

Table S2 Bonferroni corrected confidence intervals for the differences of the means of the lineages at different stages in ICM organoids and mouse blastocysts. Confidence intervals that contain zero, are highlighted in gray.

	48 h ICM organoids vs 24 h ICM organoids (in %)	late blastocysts vs mid blastocysts (in %)
DN	[-13.0, 5.2]	[-0.23, 22.1]
DP	[-10.0, -2.8]	[-26.7, 14.2]
N+/G-	[-13.7, 6.2]	[-16.3, 7.8]
N-/G+	[2.8, 25.3]	[4.6, 23.0]

Table S3 Bonferroni corrected confidence intervals for the differences of the means of the lineages in ICM organoids and mouse blastocysts. Confidence intervals that contain zero, are highlighted in gray.

	mid blastocysts vs 24 h ICM organoids (in %)	late blastocysts vs 48 h ICM organoids (in %)
DN	[-28.6, -11.0]	[-18.1, 8.2]
DP	[1.7, 16.9]	[-7.0, -2.6]

N+/G-	[-17.5, 3.0]	[-21.5, 6.1]
N-/G+	[6.7, 28.8]	[5.9, 29.1]

Table S4 Bonferroni corrected confidence intervals for the differences of the means of the neighbor distributions in ICM organoids and mouse blastocysts. Confidence intervals that contain zero, are highlighted in gray.

	mid-blastocysts vs 24 h ICM organoids (in %)			
	DN	DP	N+/G-	N-/G+
DN	[-53.5, -40.7]	[0.7, 19.5]	[-13.3, 9.8]	[25.2, 52.2]
DP	[-7.4, -5.1]	[6.8, 12.6]	[-16.3, -10.5]	[7.2, 12.7]
N+/G-	[-16.7, -14.5]	[0.6, 5.4]	[-7.4, 0.05]	[13.1, 19.4]
N-/G+	[-13.4, -10.9]	[-0.2, 4.2]	[-5.5, -0.7]	[10.2, 16.3]

	late blastocysts vs 48 h ICM organoids (in %)			
	DN	DP	N+/G-	N-/G+
DN	[11.4, 22.1]	[-1.5, -1.2]	[-18.9, -12.6]	[-4.4, 5.1]
N+/G-	[-10.6, -2.8]	[-5.6, -5.1]	[-7.1, 2.8]	[10.0, 18.5]
N-/G+	[-6.0, -1.9]	[-3.6, -3.3]	[-7.2, -2.7]	[9.9, 14.9]
